# Pickles

**Published:** 1873-03

**Authors:** 


### PICKLES

Are supposed by many to be an exceedingly unhealthful article of diet. Two things are certain—many persons have a strong craving for them, and they create or develop an appetite. Vinegar is said to be nearest in nature to the qualities of those stomach juices which perform the offices of digestion ; hence vegetable vinegar must promote, must aid digestion ordinarily, and in such cases, when no ill effect seems to follow, must do good.

Feverish persons often crave pickles, something sour—that is, the system needs an acid ; acids are found to promote the secretion of bile. We may infer, then, that when a person craves something sour, it is nature's instinct calling for a remedy for fever or biliousness or indigestion. It is the vinegar in the pickles which does the good ; the article pickled, whether it be a cucumber or onion, or any other vegetable, is as worthless as nutriment as a piece of dried wood. The cucumber, as a pickle, is peculiarly appropriate from its spongy nature ; by soaking it in salt water its own juices are absorbed by the salt in part, but in greater part displaced, as salt water is heavier than the juice of the vegetable and presses it out, pushes it aside ; the salt water being more dense than this natural juice the vegetable shrivels. But when these shrivelled, salted pickles, in the process of pickling, are put into fresh water and washed of their saltness, the vegetable regains its natural plumpness. When thus washed and allowed to drain, the vegetable is put into strong vinegar, it absorbs that vinegar like a sponge, and is ready for the table ; the more free of salt it is, the more full of good vinegar, the better it is as a pickle. Ninety-seven per cent. of a cucumber is water, hence ninety-seven per cent. of a cucumber pickle is vinegar ; only three per cent. is solid, hence, if in eating a cucumber pickle, the solid part is swallowed, it is too minute in quantity to interfere with any ordinary stomach, even if it is weak or dyspeptic, hence the idea that pickles are unhealthful as an article of diet, except in the few cases where discomfort of some kind invariably follows their consumption, is without foundation. Not only so, but pickles aid digestion, and when "craved" are a positive benefit as it is nature dictating the use of an article which she requires at the time, either to supply a natural want, or as a means of antagonizing an unnatural or diseased condition of the system. Therefore, to persons who like or crave pickles, they are healthful when properly made with vegetable vinegar.

